# Remote-controlled cholangiography injection device: first clinical study in China

**DOI:** 10.1186/s12876-021-02087-8

**Published:** 2022-01-07

**Authors:** Huan Chen, Li-yu Shan, Tao Ma, Yue Wang, Zhe Feng, Ding-Hui Dong, Yi Lv, Hao-Yang Zhu

**Affiliations:** 1 National Local Joint Engineering Research Center for Precision Surgery and Regenerative Medicine, First Affiliated Hospital of Xi’an Jiaotong University, Xi’an, 710061 China; 2 Department of Anesthesiology, First Affiliated Hospital of Xi’an Jiaotong University, Xi’an, 710061 China; 3 Department of Hepatobiliary Surgery, The First Affiliated Hospital of Kunming Medical University, Kunming, 650032 China; 4 Department of Hepatobiliary Surgery, First Affiliated Hospital of Xi’an Jiaotong University, Xi’an, 710061 China

**Keywords:** Cholangiography, Equipment design, Remote control technology, Injection device, Occupational exposure

## Abstract

**Background:**

X-ray cholangiography is of great value in the imaging of biliary tract diseases; however, occupational radiation exposure is unavoidable. Moreover, clinicians must manually inject the contrast dye, which may result in a relatively high incidence of adverse reactions due to unstable injection pressure. Thus, there is a need to develop a novel remote-controlled cholangiography injection device.

**Methods:**

Patients with external biliary drainage requiring cholangiography were included. A remote-controlled injection device was developed with three major components: an injection pump, a pressure sensor, and a wireless remote-control panel. Image quality, adverse reactions, and radiation dose were evaluated.

**Results:**

Different kinds of X-ray cholangiography were successfully and smoothly performed using this remote-controlled injection device in all patients. The incidence of adverse reactions in the device group was significantly lower than that in the manual group (4.17% vs. 13.9%, *P* = 0.001), and increasing the injection pressure increased the incidence of adverse reactions. In addition, the device helped operators avoid ionizing radiation completely.

**Conclusions:**

With good control of injection pressure (within 10 kPa), the remote-controlled cholangiography injection device could replace the need for the doctor to inject contrast agent with good security and effectivity. It is expected to be submitted for clinical application.

## Background

Biliary tract diseases are common in hepatobiliary surgery, with the incidence of gallstones alone reaching up to 10% [1]. In addition, the biliary tract is complicated, with anatomical variations present in approximately 35% of patients. Consequently, there are great challenges in the diagnosis and treatment of biliary tract diseases [2–4]. In clinical work, the diagnosis of biliary tract diseases depends on the use of a variety of imaging examinations [5], including B-mode ultrasonography [6], computed tomography (CT) [7, 8], magnetic resonance cholangiopancreatography (MRCP) [9, 10] and X-ray cholangiography [11]. Among them, X-ray cholangiography, wherein the biliary tract is injected with a medical contrast agent under X-ray to reveal lesions, is of great value [12, 13]. Compared with other biliary display technologies, cholangiography is simpler, more convenient, and cheaper and provides real-time kinematic imaging, which other imaging modalities cannot.

However, traditional cholangiography must be performed under X-ray, and a lead suit cannot fully protect the operator against radiation [14, 15]. Therefore, occupational exposure to radiation remains a detrimental side-effect. Additionally, radiation of unprotected areas such as the eyes and hands could cause cataracts, skin damage, or even cancer [16].

Furthermore, cholangiography requires the clinician to inject contrast dye into the biliary tract; therefore, the injection speed and injection pressure are controlled manually, and the intrabiliary pressure is not monitored. Consequently, it is difficult to maintain a consistent injection speed and pressure, which could cause a high incidence of adverse reactions (5–13% in different medical centers) [17].

To solve the aforementioned problems, our team developed a novel remote-controlled cholangiography injection device, the feasibility of which has been shown in animal experiments [18]. In this study, we report the successful application of this device in the clinic. The purpose of this study was to verify the safety and efficacy of the device in clinical applications.

## Methods

### Study design and patients

**Fig. 1:**
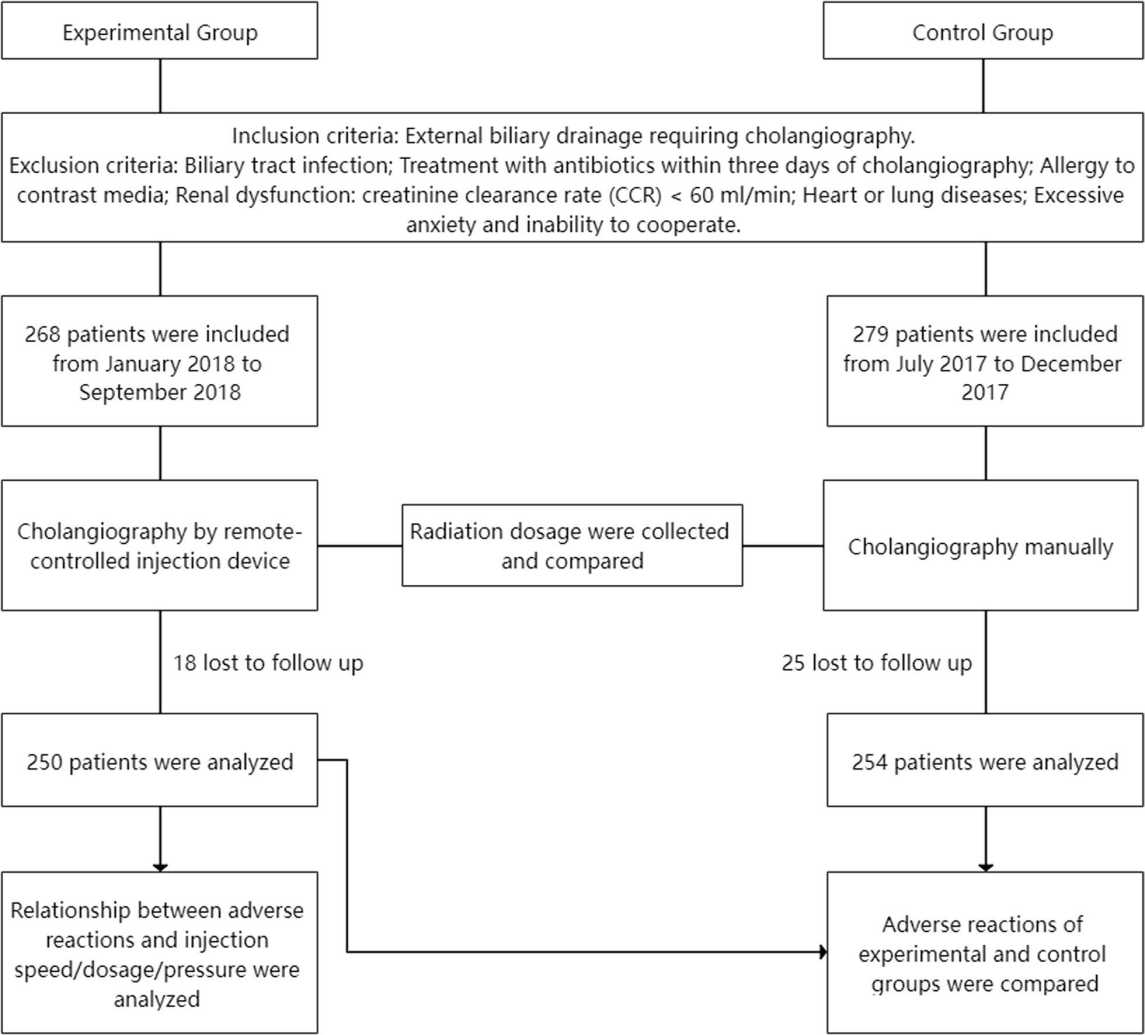
Flow diagram of the study

The design of this study is shown in Fig. 1. From January 2018 to September 2018, 268 patients with percutaneous biliary tubes who needed cholangiography were selected from the First Affiliated Hospital of Xi'an Jiaotong University to undergo cholangiography using our remote-controlled cholangiography injection device. From July 2017 to December 2017, 279 patients with percutaneous biliary tubes undergoing traditional cholangiography were chosen from the First Affiliated Hospital of Xi'an Jiaotong University as the control group.

Inclusion criteria:

1. External biliary drainage requiring cholangiography

Exclusion criteria:

1. Biliary tract infection
2. Treatment with antibiotics within three days of cholangiography
3. Allergy to contrast media
4. Renal dysfunction: creatinine clearance rate (CCR) < 60 ml/min
5. Heart or lung diseases
6. Excessive anxiety and uncooperativeness

All protocols were approved by the Ethics Committee of the First Affiliated Hospital of Xi'an Jiaotong University (NO. XJTU1AF2015LSL-046). All procedures performed in this study involving human participants were in accordance with the ethical standards of the institutional research committee and with the 1964 Helsinki Declaration and its later amendments. The study was registered on ClinicalTrials.gov (NCT02801500, 16/6/2016). Informed consent was obtained from all participants prior to cholangiography.

### Equipment

The remote-controlled injection device consists of a control terminal and an operation terminal, which communicate with each other wirelessly via Bluetooth. The structure and program were designed based on clinical operation requirements.

**Fig. 2:**
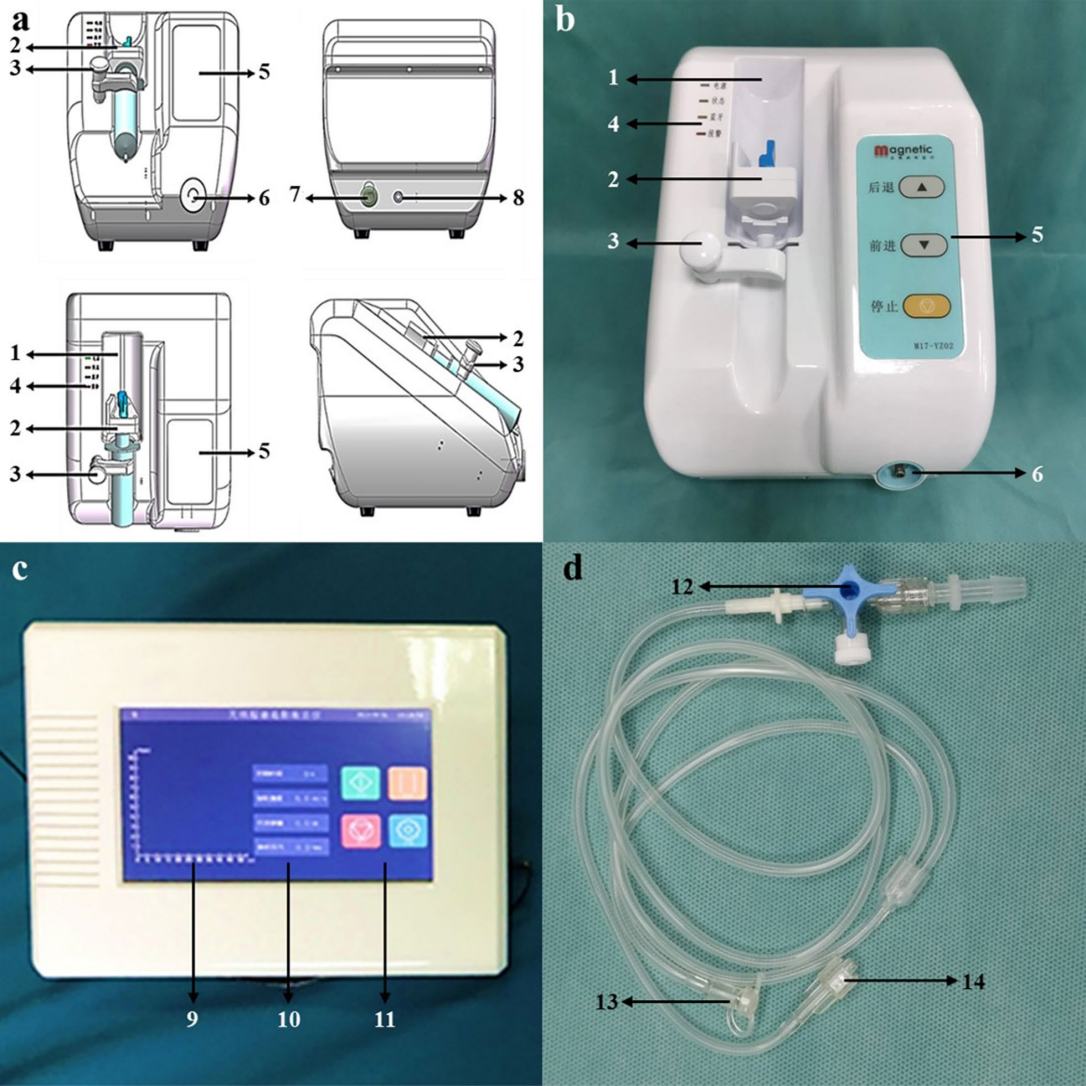
Remote-controlled cholangiography injection device. **a** Fabrication drawing. **b** Operation terminal. **c** Control terminal. **d** Extension tube. (1) Syringe slot, (2) syringe pushing hand—injection pump, (3) syringe fixing arm, (4) working status indicator, (5) direct-control panel, (6) pressure sensor, (7) on/off button, (8) power port, (9) pressure curve diagram, (10) parameter setting and display buttons, (11) control buttons, (12) T-adapter port, (13) syringe port, (14) pressure port

The control terminal consists of a wireless remote-control panel (Fig. 2c). Using this panel, operators can set up parameters (including injection speed, injection dose, and pressure threshold) and command the operation terminal. In addition, real-time parameters can be collected during cholangiography and exhibited to the operators, such as injection speed, pressure, and dose.

The operation terminal consists of an injection pump, a pressure sensor, and a direct-control panel (Fig. 2a, b). Using the remote-control or direct-control panel, the injection pump can replace a human operator for injecting the contrast dye. The pressure sensor is responsible for detecting the real-time injection pressure. Apart from the method of communication and the display of injection parameters, the functions of the direct-control panel are similar to those of the remote-control panel.

This device requires an extension tube to complete cholangiography. The extension tube has three ports: a syringe port, a pressure port, and a T-adapter port (Fig. 2d). The syringe port, pressure port, and one outlet of the T-adapter port are used to connect a 50-ml syringe, a pressure sensor, and an external biliary drainage tube separately. Another outlet of the T-adapter port can be used to connect a 20-ml syringe for degassing the biliary drainage tube.

### Procedure for the experimental group (remote-controlled cholangiography injection device group)

#### Device preparation

**Fig. 3:**
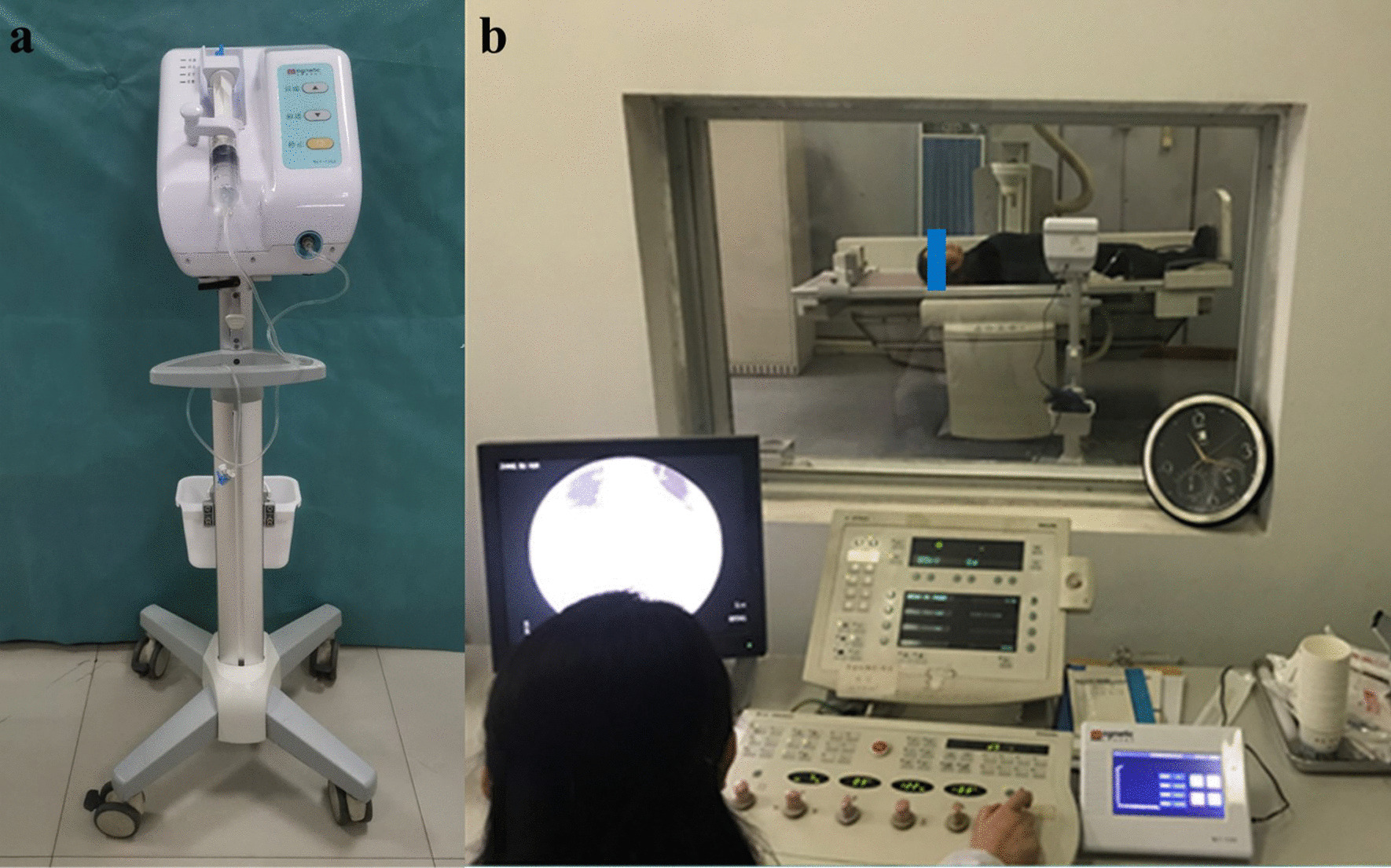
Clinical application of the remote-controlled cholangiography injection device. **a** Preparation of the device and assembly of the operation terminal, syringe and extension tube. **b** Cholangiography using the remote-controlled device

The procedure involved connecting a 50-ml syringe filled with 40 ml of diluted contrast agent (1:1, using normal saline) to the syringe port with an extension tube and prefilling the extension tube with the contrast agent. The 50-ml syringe was then loaded onto the injection pump, and the pressure port of the extension tube was connected to the pressure sensor (Fig. 3a).

#### Patient preparation

Before cholangiography, patients were required to fill out a form to facilitate post-cholangiography follow-up. The patient then entered the X-ray room and stood in front of the X-ray machine. After the machine was positioned over the right upper abdomen by X-ray, the patient was adjusted to be supine with the head high (20°). The T-adapter of the extension tube was then connected to the external biliary drainage tube (T-tube, percutaneous transhepatic biliary drainage [PTBD], etc.), and a 20-ml syringe was used to extract the remaining air bubbles in the drainage tube and biliary tract from the T-adapter via the extension tube.

### Cholangiography procedure using the remote-controlled cholangiography device

The device was adjusted to the correct settings and connected to the power supply. The operator then left the X-ray room and entered the observation room, opened the remote-control panel, and connected the parts of the device via Bluetooth. The injection speed, dose, and pressure threshold were set before initiation of cholangiography and could be adjusted according to the clarity of the image (Fig. 3b).

During cholangiography, the real-time injection speed, dose, and pressure were displayed on the remote-control panel, and the operator could stop or start the injection at any moment. If the feedback pressure surpassed the threshold, the equipment would activate an alarm and stop the injection automatically. After the cholangiography procedure, the operator connected the drainage tube and drainage bag for the patient.

### Measurement of occupational exposure

During cholangiography, radiation was monitored by three identical radiation monitors. One was completely exposed to the radiation in the X-ray room, one was protected by a lead suit in the X-ray room, and the third was exposed to natural background radiation in the observation room.

### Patient follow-up

One day after cholangiography, the patients were followed up by telephone to assess whether they had symptoms of bile duct inflammation, including fever, jaundice, and abdominal pain.

### Data collection for the control group (traditional cholangiography group)

We collected information (including demographic data, type of disease, and type of surgery) from patients undergoing traditional cholangiography from July to December 2017 at the First Affiliated Hospital of Xi'an Jiaotong University. We followed up these patients via telephone to assess whether there were post-cholangiography complications.

### Statistical analysis

SPSS Statistics Software 23.0 (IBM Corporation, Armonk, NY, USA) was used for all analyses. Categorical variables are reported as numbers and proportions and were compared using chi-square or nonparametric tests as appropriate. Continuous variables are reported as the means ± SD or medians (interquartile range (IQR)) and compared using the t test, ANOVA, or nonparametric tests. All hypothesis tests were two-sided, and *P* values < 0.05 were considered statistically significant.

## Results

### Demographic data

From January 2018 to September 2018, 268 patients from the First Affiliated Hospital of Xi'an Jiaotong University underwent cholangiography using our remote-controlled cholangiography injection device, 18 of whom were lost to follow-up. From July 2017 to December 2017, 279 patients from the First Affiliated Hospital of Xi'an Jiaotong University underwent traditional cholangiography, 25 of whom were lost to follow-up. The demographic data of these two groups are shown in Table 1. We found no significant differences in demographic variables such as age, sex, and disease.

**Table 1:** Demographic data of the experimental and control groups

	Experimental group	Control group	*P* value
Age (mean ± SD/years)	58.14 ± 14.25	56.55 ± 14.21	0.211
Sex (male/female)	127/123	120/134	0.425
Classification of diseases			
Biliary Calculi (male/female)	81/95	90/115	0.678
Hepato-Bilio-Pancreatic Cancer (male/female)	18/13	15/9	0.739
Liver transplantation (male/female)	19/4	11/3	> 0.999
Others* (male/female)	11/10	5/6	0.710

*Others include patients who underwent a second operation, choledochojejunostomy, etc.

### Image quality

**Fig. 4:**
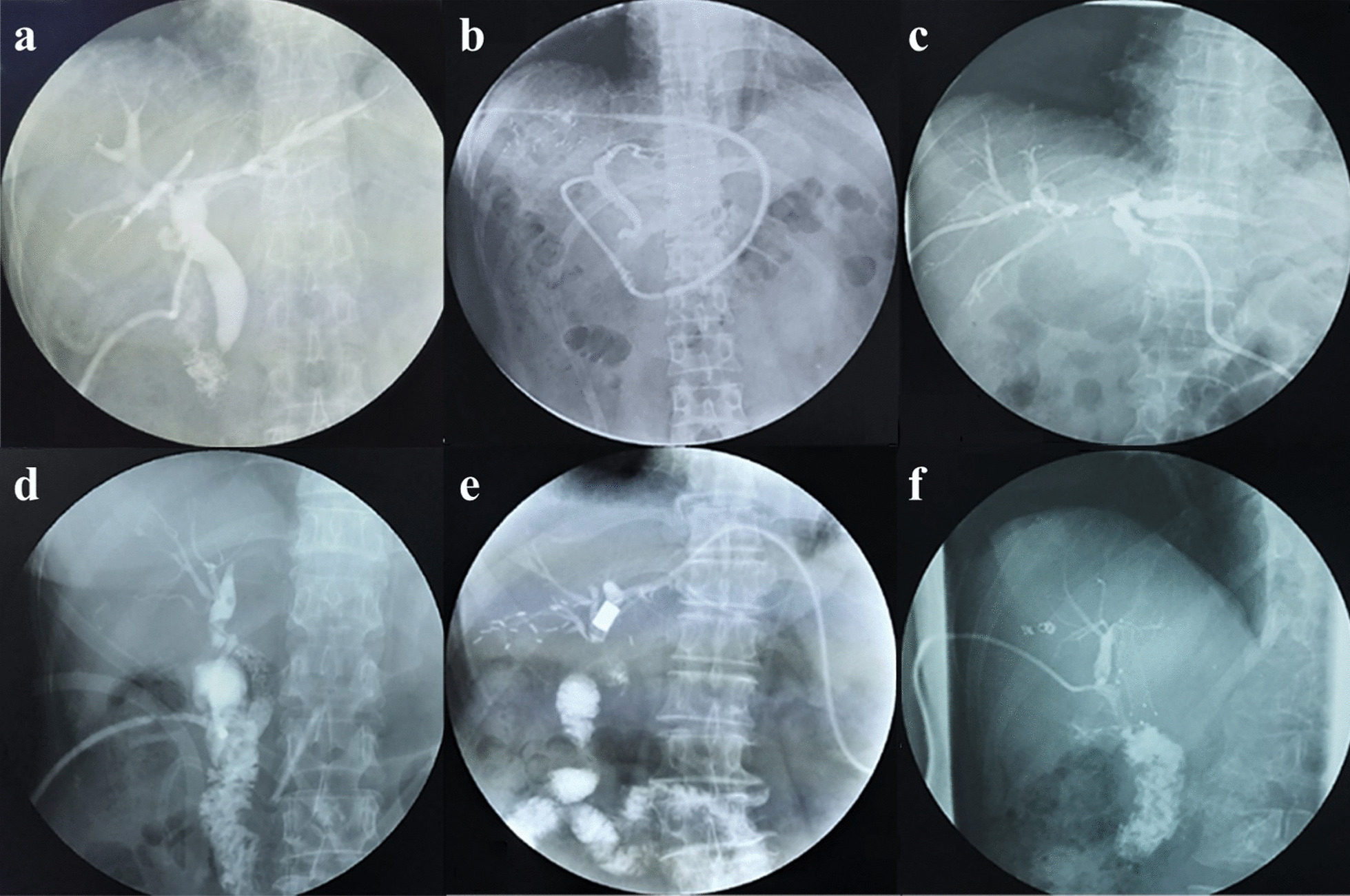
Results of remote-controlled cholangiography. **a** Normal biliary system. The common bile duct, intrahepatic bile duct and part of the intestinal mucosa are exhibited. **b** Right hemihepatectomy. The common bile duct and left hepatic duct are well visualized. **c** Hilar cholangiocarcinoma. The left and right hepatic ducts and their branches can be fully visualized through PTCD. The common hepatic duct, common bile duct and duodenum are not visualized. **d** Multiple biliary calculus. Multiple filling defects of the biliary system are displayed. Dilation and filling defects of the right hepatic duct can be seen. **e** Magnetic compressing biliary-intestinal anastomosis. The bile duct was connected to the intestine by a pair of magnets. The biliary system and intestinal mucosa can be clearly seen. **f** Postorthotopic liver transplantation. The biliary system is visualized well, and the anastomosis of the common bile duct is unobstructed

Our device successfully replaced the need for a doctor to inject the contrast agent. Different kinds of cholangiography (T-tube, PTBD, endoscopic nose biliary drainage [ENBD], etc.) using this novel remote-controlled device were successfully performed in all patients. Environmental ionizing radiation did not interfere with the remote wireless transmission, which remained stable. After contrast dye injection, the biliary system was clearly displayed on the computer screen to a degree sufficient for clinical diagnosis (Fig. 4). The procedure was smooth, and all 268 patients cooperated well.

### Adverse reactions

Ten of the 250 patients in the experimental group had mild postoperative adverse reactions, including fever and abdominal distension. In the control group, 254 patients were followed up by telephone, 31 of whom reported mild postoperative adverse reactions, including fever and abdominal distension. The incidence of adverse reactions in the experimental group was significantly lower than that in the control group (4.17% vs. 13.9%,* P* = 0.001) (Table 2).

**Table 2:** Adverse reactions of the experimental and control groups

Diseases	Groups	Numbers		*P* value
		With adverse reactions	Without adverse reactions	
Biliary Calculi	Experimental group	8	168	0.008
	Control group	25	180	
Hepato-Bilio-Pancreatic Cancer	Experimental group	1	30	0.086
	Control group	4	20	
Liver transplantation	Experimental group	1	22	> 0.999
	Control group	1	13	
Other	Experimental group	0	21	0.111
	Control group	2	9	
Total	Experimental group	10	240	0.001
	Control group	31	223	

To explore the factors affecting adverse reactions after cholangiography, we analyzed the relationship between the adverse reactions and injection parameters (including speed, dose, and pressure) in the experimental group. We found that the injection pressure had a significant effect on the occurrence of postoperative complications (Table 3). With increasing injection pressure, the incidence of adverse reactions also increased (injection pressures: 0–3 kPa; 3.1–7 kPa; 7.1–10 kPa; > 10 kPa; incidence: 1.52%; 1.41%; 10.0%; 22.2%, respectively; *P* < 0.001). In contrast to the injection pressure, when the injection speed was 0.1–0.3 ml/s, the incidence of adverse reactions was higher than in the other two high injection speed groups (injection speeds: 0.1–0.3 ml/s; 0.4–0.7 ml/s; 0.8–1.0 ml/s; incidence: 11.9%; 2.24%; 3.13%, respectively; *P* = 0.035) (Table 3). However, further analysis revealed that when the injection speed was 0.1–0.3 ml/s, the injection pressure used for all five patients who experienced adverse reactions was greater than 10 kPa. The injection dose was not a risk factor for adverse reactions.

**Table 3:** Relationship between adverse reactions and injection parameters

Groups	Numbers		*P* value
	With adverse reactions	Without adverse reactions	
Injection pressure (kPa)			
0–3	2	132	< 0.001
3.1–7	1	70	
7.1–10	1	10	
> 10	6	27	
Injection speed (ml/s)			
0.1–0.3	5	42	0.035
0.4–0.7	3	134	
0.8–1	2	64	
Injection dose (ml)			
< 20	2	57	0.618
20–30	5	84	
> 30	3	99	

### Radiation

The radiation doses from three different sources were collected, including natural background radiation (R_0_), radiation from cholangiography of exposed areas (R_1_) and radiation from cholangiography of protected areas (R_2_). The differences between the doses for these three kinds of radiation were significant (R_0_ vs. R_1_ vs. R_2_; 0.01 [IQR 0.00, 0.02]; vs. 0.16 [IQR 0.07, 0.16]; vs. 3.09 [IQR 1.67, 4.85] μSv, respectively; *P* < 0.001). The radiation dose detected in the observation room was significantly lower than that detected in the zone protected by the lead suit.

## Discussion

Cholangiography is an important examination in biliary diseases. However, it must be performed under X-ray, and occupational exposure for operators is unavoidable [14–16, 19, 20]. Additionally, cholangiography requires the clinician to manually inject contrast dye into the biliary tract. As a result, the injection speed and pressure can be variable, and the intrabiliary pressure is not monitored, which could lead to adverse reactions [21]. The rapid development of remote-controlled technology has led to its wide application in clinical practice, including in remote-controlled injection systems [22–24] and remote-controlled vascular interventional robots [25–27]. However, studies on remote-controlled cholangiography devices are scarce. In this study, a remote-controlled cholangiography injection device was developed and applied in the clinic. The feasibility, security, and efficacy of this device were verified.

In this study, we analyzed and quantified two major defects of traditional cholangiography by collecting clinical data, including occupational exposure and adverse reactions. For occupational exposure, we found that although wearing a lead suit could reduce the radiation dose significantly (R_1_ vs. R_2_; 0.16 [IQR 0.07, 0.16] vs. 3.09 [IQR 1.67, 4.85] μSv; *P* < 0.001), it was unable to eliminate occupational exposure completely (R_0_ vs. R_1_; 0.01 [IQR 0.00, 0.02] vs. 0.16 [IQR 0.07, 0.16] μSv; *P* < 0.001). Furthermore, wearing a heavy lead suit undoubtedly increases the workload and affects the operation. We therefore proposed that to eliminate occupational exposure, remote-control measures must be adopted to replace the wearing of a lead suit. In this study, the Bluetooth signal of the remote-controlled cholangiography injection device could be transmitted stably in the X-ray environment and assisted in performing remote-controlled cholangiography sensitively, accurately, and without delay. Therefore, this device could satisfy the technical requirements of cholangiography and avoid the radiation exposure from traditional cholangiography. Additionally, we believe that the surgical operation will not affect the functioning of this equipment. Thus, intraoperative cholangiography can be realized both in open surgery and laparoscopically, and the only requirement is that the extension tube must be kept sterile.

Due to the anatomical relationship between the bile duct and the hepatic sinus, when biliary pressure increases, bile-blood reflux occurs [28]. If there are bacteria in the bile, they would reflux into the blood and could cause bacteremia [29]. However, in traditional cholangiography, the injection pressure is manually controlled and was not monitored. Therefore, fever and chills after cholangiography are common [17]. In this study, the incidence of adverse reactions in the experimental group was significantly lower than that in the control group (4.17% vs. 13.9%, *P* = 0.001). In the experimental group, the contrast dye was injected by a machine, the injection speed was stable, and the injection pressure was monitored. Therefore, an acute increase in the injection pressure was prevented. The existence of a pressure threshold further assisted in avoiding an acute increase in the injection pressure, which was difficult in the control group. Thus, in the experimental group, the incidence of adverse reactions could be reduced relative to that of traditional cholangiography.

We analyzed the relationship between adverse reactions and injection parameters (including speed, dose, and pressure) collected by our remote-controlled device and found that injection pressure had a significant effect on the occurrence of postoperative complications. With increasing injection pressure, the incidence of adverse reactions also increased. Of the 10 patients who experienced adverse reactions after cholangiography, eight had injection pressures of more than 10 kPa. In contrast to the injection pressure, when the injection speed was 0.1–0.3 ml/s, the incidence of adverse reactions was higher than in the other two high injection speed groups. However, further analysis revealed that when the injection speed was 0.1–0.3 ml/s, the injection pressure used for all five patients who had adverse reactions was greater than 10 kPa. This further confirmed that high injection pressure was a proximate cause of adverse reactions, while the injection speed could influence postoperative complications by affecting the injection pressure. Good control of the injection pressure can reduce adverse reactions associated with cholangiography. In this study, we used a pressure sensor that displayed the real-time injection pressure during cholangiography, allowing the operator to adjust the injection speed according to the changing injection pressure. We proposed that to reduce postoperative complications in cholangiography, the injection pressure should be controlled to within 10 kPa.

This study has several limitations that need to be mentioned here. First, this research was limited to a single center, and further large and multi-institutional research is needed. Second, because of the use of an extension tube, the obtained injection pressure was not the exact pressure in the bile duct, although the device could accurately indicate the change in pressure in the bile tract. Third, although the study was of a prospective nature, the learning and trial periods of the device delayed the research on the experimental group. Thus, the control and experimental groups were investigated at different times, potentially resulting in bias or misunderstanding.

## Conclusions

In conclusion, clinical application proved that the remote-controlled cholangiography injection device could clearly display the biliary system structure, eliminate occupational exposure for the operator, and reduce adverse reactions by controlling the injection pressure to within 10 kPa. The device is the first remote-controlled cholangiography system in China. The device could replace traditional cholangiography and is expected to be of value in the clinic.

## Data Availability

The clinical datasets generated and analyzed during the study are not publicly available due to privacy issues but are available from the corresponding author on reasonable request.

## Abbreviations

ANOVA: Analysis of variance
CCR: Creatinine clearance rate
CT: Computed tomography
ENBD: Endoscopic nose biliary drainage
IQR: Interquartile range
MRCP: Magnetic resonance cholangiopancreatography
PTBD: Percutaneous transhepatic biliary drainage
